# Current progression in application of extracellular vesicles in central nervous system diseases

**DOI:** 10.1186/s40001-023-01606-5

**Published:** 2024-01-03

**Authors:** Xiang-Min Zhang, Jie Huang, Xiao-Ying Ni, Hui-Ru Zhu, Zhong-Xin Huang, Shuang Ding, Xin-Yi Yang, Yan-Di Tan, Jian-Fu Chen, Jin-Hua Cai

**Affiliations:** 1 Department of Radiology Children’s Hospital of Chongqing Medical University, National Clinical Research Center for Child Health and Disorders, Ministry of Education Key Laboratory of Child Development and Disorders, No. 136, Zhongshan Second Road, Chongqing, 400014 China; 2 Chongqing Engineering Research Center of Stem Cell Therapy, No. 136, Zhongshan Second Road, Chongqing, 400014 China; 3 Department of Ultrasound the Third Affiliated Hospital of Chongqing Medical University, No. 1, Shuanghu Branch Road, Huixing Street, Chongqing, 401120 China; 4 Department of Ultrasound, The Second People’s Hospital of Yunnan Province, No. 176, Qingnian Road, Kunming, 650021 China

**Keywords:** Extracellular vesicles, Central nervous system diseases, Image, Molecular therapy

## Abstract

Early diagnosis and pharmacological treatment of central nervous system (CNS) diseases has been a long-standing challenge for clinical research due to the presence of the blood–brain barrier. Specific proteins and RNAs in brain-derived extracellular vesicles (EVs) usually reflect the corresponding state of brain disease, and therefore, EVs can be used as diagnostic biomarkers for CNS diseases. In addition, EVs can be engineered and fused to target cells for delivery of cargo, demonstrating the great potential of EVs as a nanocarrier platform. We review the progress of EVs as markers and drug carriers in the diagnosis and treatment of neurological diseases. The main areas include visual imaging, biomarker diagnosis and drug loading therapy for different types of CNS diseases. It is hoped that increased knowledge of EVs will facilitate their clinical translation in CNS diseases.

## Introduction

Extracellular vesicles (EVs) constitute one of the communication pathways between cells, ranging in size from 40 to 10,000 nm. They are classified into three subtypes: microvesicles, exosomes, and apoptotic bodies [1]. As messengers facilitating signal transmission between cells, EVs participate in various molecular responses by promoting the transport of proteins, lipids, mRNA, miRNA, and DNA. They play a crucial role in promoting neural development, regulating the progression of inflammation, and altering tumor characteristics [2–4] Alterations in EVs signaling, especially changes in miRNA expression, can indicate variations in the physiological microenvironment, closely associated with the degree of central nervous system (CNS) injury [5]. EVs associated with CNS diseases can traverse the blood–brain barrier (BBB) and participate in peripheral blood circulation [6, 7]. Extracting EVs from peripheral blood for early diagnosis, progression monitoring, and prognosis assessment of CNS diseases is considered a safer and more sensitive non-invasive method compared to “cerebrospinal fluid biopsy” [8–10]. Therefore, EVs, serving as biomarkers, reflect changes in the CNS microenvironment, offering new insights for the diagnosis and treatment of CNS diseases [11].

In addition, they can serve as carriers to protect their contents from immune system engulfment and enhance the accumulation of substances of interest within specific regions, such as contrast agents and drugs [12–14]. The BBB relies on the neurovascular unit, composed of endothelial cells, pericytes, perivascular fibroblasts, astrocytes, microglia, and nerve terminals, to maintain and develop [15, 16]. It can protect the CNS from harmful substances of blood. Furthermore, ATP-binding cassette transporters are extensively present in the BBB, facilitating the reverse transport of various substances into the bloodstream outside the CNS. This results in the low bioavailability of drugs in CNS diseases [17]. Therefore, the BBB restricts the imaging monitoring or drug efficacy in CNS diseases [18, 19]. Although other exogenous nanomaterials can also cross the BBB for drug transport, the uncontrollable risks posed by their in vivo accumulation and blood toxicity have yet to be effectively addressed.

EVs with a diameter of approximately 30–150 nm are also referred to as exosomes [20]. These EVs are a group of multivesicular bodies mediating intercellular communication. They are formed by invagination of lysosomal particles and released into the extracellular matrix by fusion of the multivesicular bodies outer membrane with the cell membrane [21, 22]. The tetrapeptides (CD9, CD63 and CD81), lectins, integrins, intracellular adhesion molecules and proteoglycans in EVs synergistically enhance endocytosis of recipient cells to facilitate cell signaling [23–27]. Small nanoscale EVs not only achieve targeted drug transport across the BBB by specific binding to brain microvascular endothelial cells through surface ligands but also possess advantages of low toxicity, low immunogenicity, high biocompatibility, and high stability. EVs have the inherent ability to stably transport cargos to target cells and perform functions [28]. After engineering modification of the EVs surface, EVs can be induced to target specific tissues [29, 30], offering great advantages for imaging and treatment of CNS diseases [31].

In this review, we provide an overview of the applications of EVs as biomarkers and nanocarriers in the field of CNS diseases, covering three aspects: diagnosis, imaging, and treatment. Particularly in the realm of therapeutic research, we have conducted a comprehensive review of five different types of CNS diseases: cerebral ischemia, glioma, Parkinson's disease (PD), depression, and Huntington’s disease (HD). These diseases were chosen, because they represent common and challenging vascular diseases, malignant tumors, neurodegenerative diseases, psychiatric disorders, and genetic disorders of the CNS, respectively. We aim to enhance awareness of EVs research in CNS diseases, thereby driving further exploration and advancement of EVs in this field.

## Imaging

In the past, researchers used fluorescent dyes or radioactive elements to label EVs, and observed the circulation pathway of EVs through imaging [32–34]. Recent studies have demonstrated [35–38], that EVs can also be loaded with contrast agents to enable molecular imaging and visualize lesions, including: superparamagnetic iron oxide nanoparticle (SPION) and reporter genes. SPION of approximately 5 nm to 150 nm, consists of magnetic iron oxide in crystalline form [39]. Based on stability considerations, a sufficiently small contrast agent needs to be selected for binding to EVs, and SPION of 5 nm to 7 nm is considered to be the best choice for EVs labeling [35, 40]. Busato et al. [35] labeled adipose stem cell-derived EVs through co-culture with cells using superparamagnetic iron oxide nanoparticles (SPION). In their study, they observed a low limit of detection for magnetic resonance imaging (MRI) to be 3 μg and 5 μg, respectively, through in vitro cellular imaging and in vivo injection imaging under mouse muscle.. The labeling principle may be that SPION undergoes a series of cellular physiological processes of endocytosis and release before being excreted by EVs encapsulation [41]. Meanwhile, Hu et al. [39] loaded SPION by disrupting the pore formation of the stable bilayer of EVs through electroporation. They then applied this technique to lymph node imaging in a mouse model of melanoma. However, during co-incubation, the number of SPION decreases with cell division thus reducing the efficacy of SPION labeling of EVs, and the damage to the EVs membrane caused by electroporation may also affect EVs function. Ferritin heavy chain (FTH1) bridges this gap as a reliable reporter gene for MRI molecular imaging. After lentiviral transfection of cells with the FTH1 gene, the transgene can be continuously expressed in daughter cells [42]. This provides a theoretical basis for the sustainability of daughter cell secretion of rich FTH1-EVs. However, the low content of FTH1 leads to unsatisfactory imaging results, and FTH1-EVs may become a favorable tool for molecular imaging in MRI if methods to enhance FTH1 expression in EVs can be explored.

**Fig. 1:**
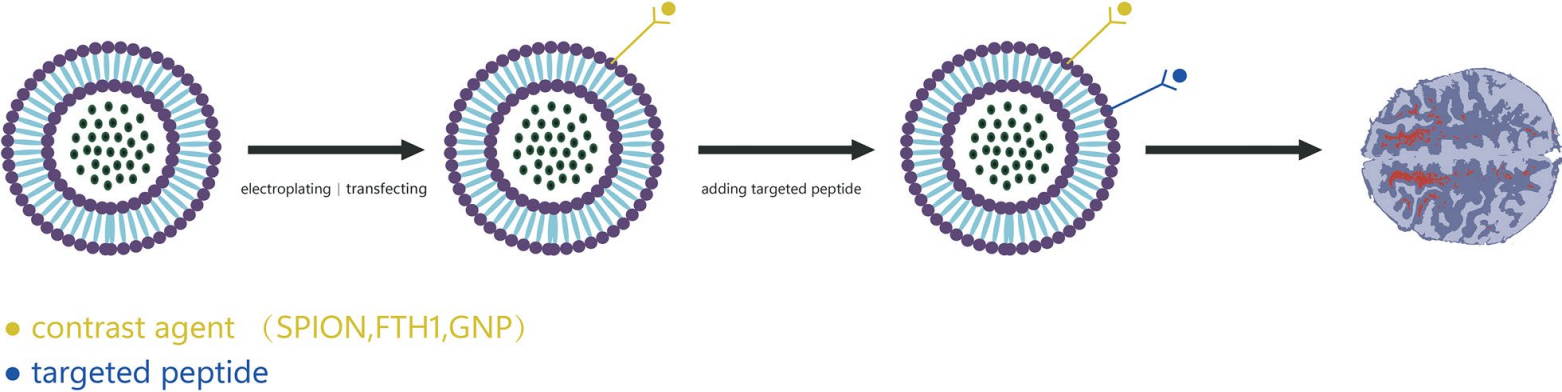
Strategies for EVs imaging. EVs are electroporated or transfected to carry contrast agents, and then chemically modified with the corresponding targeting peptide. Region of interest is imaged by crossing BBB via blood circulation

Studies in vitro have shown [43–45], that EVs isolated from bone-marrow-derived mesenchymal stem cells(BMSCs) can accumulate in prostate cancer cell lines, breast cancer cell lines, sarcoma cell lines, and gastric cancer cell lines. However, compared to stem-cell-derived EVs, tumor-cell-derived EVs showed significantly higher accumulation in colon cancer tumors at 18 h by single-photon emission computed tomography [46]. Tumor-cell-derived EVs has a high affinity to tumors and the potential mechanism of this homing phenomenon may be related to the different integrin expression patterns [47]. Besides tumor homing properties, pluripotent stem-cell-derived EVs labeled with SPION, monitored by MRI were found to accumulate in damaged and ischemic tissues [48]. Most studies take advantage of the specific targeting of EVs to certain lesions for imaging. To expand the medical applications of EVs, most EVs are gifted with stronger targeting effects mainly through engineering modifications. In particular, bioorthogonal chemistry, as a strategy for rapid modification of EVs’ surfaces, allows the conjugation of various ligands within 24 h [49–51]. By this method, the targeting peptide can bind to all EVs isolated from the culture medium or body fluids. Previous research [52–55] demonstrated a substantial increase in EVs signals in intracranial lesions following targeted peptide engineering modifications. Notably, no observable morphological or functional tissue damage was reported in the liver and lung, despite being the primary target organs enriched for EVs. Jia et al. [56], adhered neurocilin-1 to the surface of EVs by click chemistry, the primary technique of bioorthogonal chemistry, and then SPION and curcumin (Cur) were integrated into the EVs to target gliomas. In addition to tumor imaging, it can produce the dual therapeutic effects of magnetic fluid hyperthermia and chemotherapy. Glucose-coated encapsulated gold nanoparticle(GNP) can also be absorbed into EVs via glucose transporter type 1-mediated energy-dependent mechanisms, and then applied to CT(Computed Tomography)CT imaging in a focal cerebral ischemia model [57]. The ability of EVs to track infarct tissue via CT scanning has significantly advanced translation of EVs in imaging, facilitating high-resolution, sensitive and non-invasive tracking of nanomedicines [58]. The strategy for EV imaging is shown in Fig. 1.

Unfortunately, research based on EVs imaging has primarily focused on animal models, such as near-infrared fluorescence imaging. However, this imaging method is not practical for clinical applications. Therefore, this paper primarily discusses materials, such as SPION, FTH1, and GNP that have either been clinically applied or show potential for clinical use. In addition, despite numerous studies on EVs for treating CNS diseases in recent years, research on imaging the CNS is relatively scarce. However, monitoring the progression of CNS diseases by loading imaging materials is also a very promising research avenue, If EVs can carry drugs to treat CNS diseases.

## Biomarkers and therapy

**Fig. 2:**
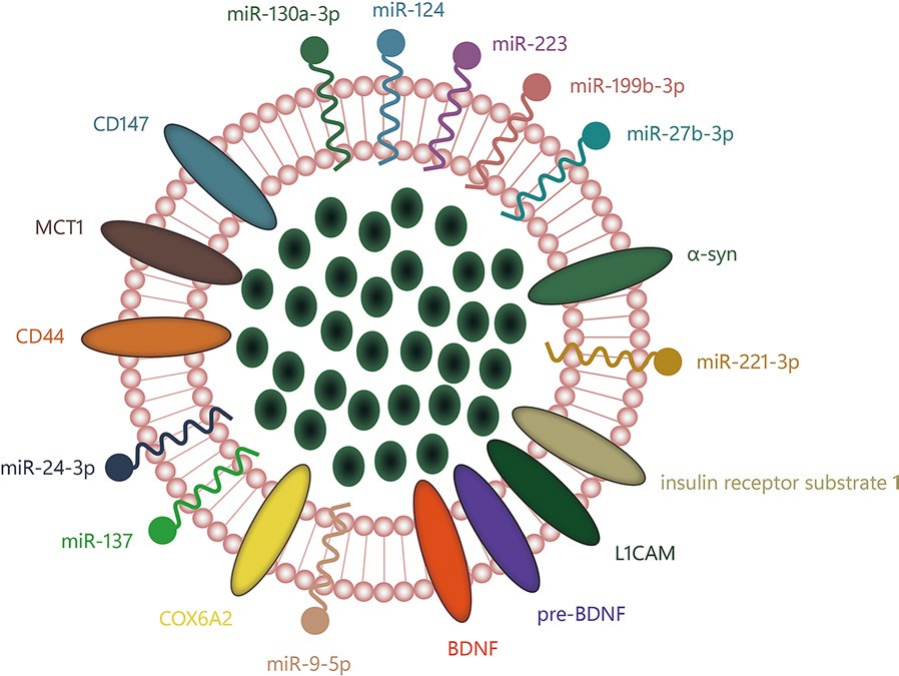
EVs carry a variety of information, such as proteins and RNA, are protected by a phospholipid bilayer, and are not easily degraded in the circulation. It can effectively respond to CNS disease progression

EVs play a crucial role in the pathophysiology of CNS diseases. In the occurrence of intracranial lesions, the levels of proteins and RNA in EVs secreted by relevant cells, including neurons and microglia, undergo corresponding changes. Since these EVs secreted by cells can freely cross the BBB, their isolation from peripheral blood allows for batch detection and analysis through methods, such as western blotting, enzyme linked immunosorbent assay, flow cytometry, quantitative real-time polymerase chain reaction, or nanoparticle tracking analysis [59]. As shown in Fig. 2, EVs are protected by a lipid bilayer, making it difficult for the enzymes to degrade. EVs are superior to unprotected circulating DNA or RNA, providing real-time information for early diagnosis and disease progression. They serve as sensitive biomarkers, responding promptly to CNS diseases [60–62].

In addition, due to their small size, low immunogenicity, high delivery efficiency, and strong biocompatibility, EVs can enter brain endothelial cells through the following pathways: membrane fusion with target cells, receptor–ligand interaction-driven internalization, and endocytosis [63]. Evs, carrying cargo, cross the BBB, modulating and alleviating CNS diseases. Evs serve as ideal nano-carriers for various small-molecule drugs, especially miRNA. To enhance targeting ability of Evs, they are typically subjected to engineering modification before use, representing a crucial research approach in the field of EV therapy for CNS diseases. The following information of Evs in the application of CNS is provided in Table 1.

**Table 1:** Therapeutic application of Evs in CNS

Representatives	Study	Object	Model	Sample size	Evs Source	Targeting peptide	Active ingredient	Loading method	Administration route
Cerebral ischemia	Tian [85]	C57BL/6	MCAO	*n* ≥ 8	BMSCs	c(RGDyK)	Cur	Sucrose gradient centrifugation	Tail vein injection
	Zhang [74]	Wistar rats	TBI/CCI	*n* = 40	hMSCs	–	–	–	Tail vein injection
	Li [75]	Sprague–Dawley	MCAO	*n* = 54	BMSCs	–	miR-150-5p	LNP transfection	Stereotactic injection
	Yang [81]	–	PT	*n* = 18	HEK293	RVG	circSCMH1	Plasmid transfection	Tail vein injection
	Tian [73]	C57BL/6	MCAO	*n* = 15	ReN	Arg-Gly-Asp (RGD)	–	Lentivirus transfection	Tail vein injection
	Guo [88]	SD rats	MCAO/R	*n* = 6	whole blood of SD	mAb GAP43	Quercetin	Co-incubation	Tail vein injection
Glioma	Zhang [104]	C57BL/6	GL261	*n* = 40	endothelial cells	–	Dox	Emulsion method	Tail vein injection
	Wang [105]	Nude Mouse	C6	*n* = 30	Neutrophils	–	Dox	Sonication	Tail vein injection
	Zhu [107]	Nude Mouse	U87	*n* = 20	ESCs	c(RGDyK)	PTX	Co‐incubation	Tail vein injection
	Li [108]	Nude Mouse	LN-229	*n* = 35	hMSCs	iron oxide nanoparticles (IONPs)	siGPX4/BQR	Electroporation	Tail vein injection
	Zhan [110]	Nude Mouse	TBD0220	*n* = 35	Serum of healthy animals	–	cPLA2 siRNA/metformin	Electroporation	Tail vein injection
Parkinson’s disease	Cai [127]	C57BL/6	PD	*n* = 24	BMSCs	–	Gli1	Plasmid transfection	Tail vein injection
	Peng [128]	C57BL/6	PD	*n* = 35	MSCs	RVG29	curcumin	ultrasonically Oscillated /extrusion by mini-extruder	Intranasal administration
	Kojima [129]	C57BL/6	PD	*n* = 8	hMSCs	RVG	Nanoluc mRNA	Electroporation transfection	Subcutaneous injection
	Wang [130]	C57BL/6	PD	*n* = 15	hMSCs	–	PMA	Co‐incubation	Tail vein injection
Depression	Li [159]	BALB/c	chronic mild stress	*n* = 30	Natural killer cells	–	miR-207	–	Tail vein injection
	Guo [160]	Sprague–Dawley	the injury of hippocampal neurons	*n* = 100	hMSCs	–	microRNA-26a	Lipofectamine kit	Tail vein injection
Huntington’s disease	García [164]	XBP1 flox/flox	YAC128 and R6/2	*n* ≥ 77	Neuro2a cell	–	Insulin-like growth factor 2 (IGF2)	Lipofectamine RNAiMAX kit	Stereotactic injection
	Lee[166]	HD	R6/2 line of transgenic	*n* = 22	HEK 293 cells	–	miR-124	Plasmid transfection	Stereotactic injection
	Zhang [170]	C57BL/6 J	N171-82Q, BACHD, YAC128	*n* ≥ 40	Liver cells	RVG	mHTT siRNA	Naked DNA plasmids(genetic circuits)	Tail vein injection

MCAO: Middle Cerebral Artery Occlusion; MCAO/R: focal cerebral ischemia/reperfusion; TBI: Traumatic brain injuries; CCI: controlled cortical impact; PT: photothrombotic (PT) stroke; LNP: Lipid Nanoparticle; PTX: paclitaxel; Dox: doxorubicin; Cur: curcumin; ReN: ReNcell VM; ESCs: Embryonic stem cells; BQR: brequinar; PD: Parkinson disease; PMA: poly (methacrylate arginine); MSCs: mesenchymal stem cell; BMSCs: bone marrow mesenchymal stem cells; hMSCs: human mesenchymal stem cells

## Cerebral ischemia

In trauma or other pathological conditions, brain tissue is susceptible to damage due to ischemia and hypoxia, accounting for the second highest mortality rate from disease [64]. The miR-124 and miR-223, are significantly related to stroke occurrence, infarct volume and prognosis, and miR-199b-3p, miR-27b-3p, miR-130a-3p, miR-221-3p and miR-24-3p in EVs also respond to the development of stroke caused by asymptomatic carotid stenosis [65–69]. The detection of the ischemic penumbra is a critical factor whether reperfusion treatment is decided in acute ischemic stroke (AIS). Compared with the control group, CircOGDH in EVs of AIS mice were highly expressed in plasma and ischemic penumbra, increasing 54-fold and positively reflecting the size of ischemic penumbra [70]. The expression of these EVs biomarkers provides multiparametric information for the diagnosis and monitoring of ischemic encephalopathy and can also provide valuable clues to the cause of ischemia. The miRNAs in these Evs serve not only as reliable biomarkers but also as potential therapeutic drugs.Enriched with miR-132, Evs serve as potential therapeutic tools, aiming to enhance endothelial cell survival in cerebral ischemia or improve the efficiency of endothelial cell transplantation therapy for restoring blood oxygen circulation [71]. Despite these efforts, cerebral ischemia–reperfusion injury remains an unavoidable challenge [72].By inhibiting reactive astrocyte proliferation and microglia activation to alleviate inflammation-induced neurodegeneration, Evs containing miR-21, miR-125b and miR-145 were isolated from human neural progenitor cell lines to deal with inflammatory damage brought about by cerebral ischemia–reperfusion injury [73, 74]. Enrich miRNAs in neuron-derived Evs play a positive role in cerebral ischemia treatment, but the difficulty of neuronal cells to acquire large amounts of Evs by cell division may limit the clinical application of Evs therapy. BMSCs-derived Evs also have a positive effect on promoting angiogenesis, anti-apoptosis and anti-inflammation in cerebral ischemia without such strict limitations as neuronal cells. BMSCs-derived Evs enriched with miR-150-5p can silence toll-like receptor 5 to ameliorate the neuronal apoptosis activated by ischemia–reperfusion [75]. With the downregulation of toll-like receptor 5, infarct foci and edema were reduced, and neurological function was significantly restored [76, 77]. Another study also showed [78] that BMSCs-derived Evs inhibit the p38 mitogen-activated protein kinase/nuclear factor-kappaB p65 pathway to buffer polarize microglia toward M1 type (pro-inflammatory) and regulate the expression of inflammation-related transcriptional genes such as IL-1β, IL-6 and TNF-α to treat neonatal hypoxic–ischemic brain injury. To improve therapeutic efficacy, many studies have focused more on Evs engineered to enhance targeting and load small molecules for the treatment of CNS diseases. Rabies virus glycoprotein (RVG), The cyclo(Arg–Gly–Asp–D-Tyr–Lys) peptide [c(RGDyK)], and monoclonal antibody against GAP43(mAb–GAP43) included in Table 2, are commonly targeted peptides for the treatment of cerebral ischemia. The neuron-specific RVG selectively targets neuronal cells and brain endothelial cells by binding to nicotinic acetylcholine receptors [79]. Yang et al. [80], designed RVG to be localized on the surface of Evs and then loaded miR-124 within Evs can be delivered to the site of infarction to induce cortical neurogenesis. Similarly, Li et al. [81], constructed RVG–circSCMH1–Evs that specifically deliver circSCMH1 to the brain to promote functional recovery after stroke in AIS models in mice and monkeys. For ischemic diseases, targeting peptides that bind to highly expressed factors in the region of the ischemic lesion, such as c(RGDyK) and mAb–GAP43, are more well-directed. Targeting of c(RGDyK)–Evs to the ischemic areas is associated with integrin αvβ3. The angiogenic response promotes integrin avβ3 expression on reactive endothelial cells in the ischemic areas, while non-ischemic areas are normally expressed, resulting in an abundance of c(RGDyK)–Evs in the ischemic core and semidark areas [82–84]. Systematic administration of c(RGDyK)–Evs containing Cur has been shown to target ischemia areas and be taken up by microglia, neurons, and astrocytes to inhibit inflammatory responses and apoptosis [85]. Engineered Evs modified by mAb–GAP43 can be delivered to ischemic lesions by binding to GAP43 highly expressed in ischemic neurons [86]. mAb–GAP43–Evs loaded with quercetin, enhance the scavenging of reactive oxygen species(ROS) by precisely targeting ischemic lesions and activating the nuclear factor-E2 related factor-2/heme oxygenase-1 pathway to buffer nerve damage and promote neural recovery [87, 88].

**Table 2:** Targeted peptides commonly used in ischemic diseases

Targeted peptides	Receptor cells	Targeted receptors	References
RVG	Neuronal cells and endothelial cells	Nicotinic acetylcholine receptors	Lentz TL, J Mol Recognit, 1990 [79]
cRGD	Ischemic endothelial cells	Integrin αvβ3	Abumiya T, J Cereb Blood Flow Metab, 1999 [82] Li L, Exp Neurol, 2012 [83] Arosio D, Adv Drug Deliv Rev, 2015 [84]
mAb–GAP43	Ischemic neuronal cells	GAP43	Liu W, J Nanobiotechnology, 2022 [86]

## Glioma

Glioma is a fatal brain tumor with a median survival of only about 14 months even with aggressive application of surgical resection, radiation therapy and chemotherapy [89, 90]. Recently, several techniques have been developed to allow multi-parameter characterization of individual Evs, which can help isolate specific Evs subgroups for accurate disease identification. Multiparametric characterization of extracellular vesicles secreted by brain malignancies can be detected and precisely quantified by imaging flow cytometry to differentiate intracranial tumors [91, 92]. Biosensors such as localized surface plasmon resonance and atomic force microscopy can enable quantitative detection of proteins in individual Evs and observe the progression of glioblastoma and hypoxia-induced malignant gliomas at the molecular level [93, 94]. CD44 was found to be upregulated in Evs to promote glioblastoma cell migration and vascular endothelial cell formation by these techniques [93].

Furthermore, tumor cells promote tumor progression by selectively regulating EVs’ miRNAs expression [95]. It retains tumor-promoting miRNAs in vivo, accelerating tumor proliferation. Simultaneously, tumor suppressive miRNAs are isolated and delivered to immune cells in the form of Evs, reconstituting these cells into an immunosuppressive phenotype, including tumor suppressive miR-1298-5p and tumor-promoting miR-9-5p [96–100]. The synergistic effect of abundant miRNAs in glioma-derived EVs is a key mechanism that promotes tumor progression. Blocking the production and uptake of tumor-associated miRNAs in Evs to halt tumor progression is an ideal therapeutic strategy. As an excellent vehicle, EVs loaded with small molecules are likewise considered as a promising therapeutic option [101–103]. Zhang et al. [104], constructed endothelial cell-derived Evs to load doxorubicin(Dox), which were able to be taken up by endothelial cells after systematic administration of Evs–Dox, triggering apoptosis and immunogenic death in glioma cells through the following mechanisms: maturation of dendritic cells, activation of cytotoxic cells, altered cytokine production, glial glioblastoma cell proliferation inhibition and increased apoptosis. neutrophil-derived Evs has also been designed for rapid delivery of Dox drugs in a mouse model of hormonal glioma, which relies on the inherent inflammatory chemotactic properties of neutrophil-derived Evs. Studies in mice with glioma and brain inflammation [105] showed that neutrophil-derived Evs had a significant targeting effect on inflammatory stimuli induced by tumor cells, ultimately effectively inhibiting tumor growth and prolonging survival. Glioma-derived EVs loaded with temozolomide and dihydrotanshinone accumulate in tumors through tumor homologous effects and overcome resistance [106]. Various sources of Evs deliver drugs to tumors through distinct targeting mechanisms. This approach aims to maximize drug efficacy with the smallest effective dose while minimizing systemic damage caused by non-targeted drugs. Enhancing the targeting performance of Evs through artificial means can overcome limitations in Evs sources, allowing for specific drug delivery and expanding therapeutic options. Zhu et al. [107], found that specific reprogramming factors contained in EVs derived from embryonic stem cell reduced the clonogenicity and tumorigenicity of tumor cells, and loaded paclitaxel target tumor cell lines (U87 and U251) after modified with c(RGD) peptides. EVs modified with Angiopep-2 peptides are loaded with iron nanoparticles, glutathione peroxidase 4 and dihydroorotic dehydrogenase inhibitors to target glioblastoma by local magnetic localization and lipoprotein receptor protein 1 recognition. The synergy of three actions, dihydroorotic dehydrogenase catabolism, glutathione peroxidase 4 iron death defense axis and Fe^2+^ release mediated by Fe_3_O_4_ nanoparticles, promotes iron death in glioblastoma [108, 109]. The nanocomposites are stable and Fe^2+^ is released from EVs only in the tumor microenvironment under acidic conditions (pH 5.5). According to the following mechanisms: 1. Polymerase I and trans-release factor, promote Evs -uptaken by glioblastoma cells; 2. cPLA2–siRNA and metformin are highly detrimental to mitochondrial energy metabolism, personalized therapeutic regimens for polymerase I and tran release factor-EVs targeted delivery of cPLA2–siRNA/metformin have also been shown to be feasible [110].

## Parkinson’s disease

PD is one of the most common neurodegenerations with progressive degeneration of dopaminergic neurons [111, 112]. Pathological changes in nerve cells regulate substances level in EVs, reflecting PD progression. The diagnostic efficacy of α-synuclein(α-syn) in peripheral is equivalent to that of cerebrospinal fluid, and the level of α-syn in EVs isolated from peripheral blood is significantly correlated with the disease diagnosis [113]. The concentration of α-syn in Evs isolated from blood through immunoprecipitation using neuronal and oligodendroglial markers, helps identify diseases with similar symptoms, such as PD and multiple system atrophy. According to the report of Dutta et al. [114], the ratio of the α-syn concentration in EVs isolated from oligodendrocytes and neurons, was considered as helpful biomarker to identify between PD and multiple system atrophy, with a high sensitivity (89.8%) and specificity (86.0%), and area under the ROC curve measure of 0.902 in multinomial logistic model. Other proteins such as clusterin, complement C1r subcomponent, apolipoprotein A1, and fibrinogen gamma chain in plasma-derived EVs decrease to varying degrees in Yahr stage II and III, and apolipoprotein A1 level was lower in stage III than in stage II [115]. Deposition of α-syn in neuronal cells is considered to be a pathological hallmark of PD [116]. Α-syn is secreted in a way of calcium-dependent and EVs-packaged to induce mitochondrial dysfunction as well as oxidative stress, leading to cognitive impairment [117–119]. Interestingly, microglia have been shown to be involved in this process. EVs containing CD11b + secreted by microglia induce α-syn deposition in neurons [120]. Associated miRNAs and α-syn induce alterations in the genetic program of target cells, leading to PD progression through the accumulation of α-syn [121]. Nonetheless, EVs have also shown potential for tissue repair and neural regeneration, and have been successfully loaded with various drugs to retard PD [8, 122]. BMSCs-derived EVs inhibit microglia activation, accelerate the elimination of α-syn, and increase neuronal activity [123, 124]. Xu et al. [125, 126], suggested that Evs secreted during the differentiation of BMSCs into dopaminergic neurons may regulate cholesterol metabolism in the hippocampus. This regulation could potentially ameliorate PD through the Wnt5a–lipoprotein receptor protein 1 cascade. Cai et al. [127], also showed that glioma-associated oncogene homolog-1 in BMSCs-EVs inhibits leucine-rich repeat kinase 2 activation mediated by the Sp1 transcription factor promoter so as to attenuate inflammatory damage and neuronal apoptosis in PD. Moreover, MSC-EVs integrate with Cur to directly target cells and release the drug into the cytoplasm to inhibit α-syn aggregation, neuroinflammation, and neuronal damage through intranasal administration [128]. However, due to poor targeting and limited therapeutic capacity, simple EVs-based therapies are difficult to meet the therapeutic requirements of PD. To improve the efficiency of cargo delivery in EVs, Kojima et al. [129], constructed the EVs’ transfer into cells devices that promoted EVs secretion, specific miRNAs packaging, and targeted delivery to constantly transfer mRNAs to the lesions. Delivering catalase miRNAs and producing sufficient EVs through implanting the devices to save neurotoxicity and neuroinflammation caused by PD, instead of repeatedly injecting concentrated EVs in vitro, opens a new way for EVs treatment. However, whether the devices can work long-term in vivo or bring other unknown impact still needs further test and verify. The strategy of “Engineered EVs with Independent Module/Cascading Function” was proposed by Wang et al. [130, 131], guanidine groups in L-arginine derivative actively target ROS and inductible nitric oxide synthase highly expressed in the pathological microenvironment of PD, and then synthesize nitric oxide through chemical reaction. ROS and inductible nitric oxide synthase in mitochondria of impaired neurons are more than those in other healthy cells. The natural tendency between reactants is used to guide EVs into pathological cells [132, 133]. Engineered EVs repair nerve injury of PD by consuming ROS and producing nitric oxide.

## Depression

With the incidence rate increasing in adolescents, depression has received widespread attention [134, 135]. Depression is considered to be a common emotional disorder, and there is no particularly effective drug at present [136, 137]. It is well-known that neuroinflammation is closely associated with the progression of depression-like behaviors and that inflammatory drive underlies the progression of major depressive disorder(MDD) [138–140]. miR-9-5p, an MDD-related miRNAs in EVs, exacerbates neuronal inflammation and stimulates MDD progression through translocation from neurons to microglia. MiR-9-5p is highly expressed in neuronal Evs of MDD patients. It induces M1 polarization in microglia by inhibiting the expression of suppressor of cytokine signaling 2 and initiating the janus kinase/signal transducer and activator of transcription pathway. This leads to increased release of inflammatory factors, including interleukin-1β (IL-1β), interleukin-6 (IL-6), and tumor necrosis factor-α (TNF-α) [141]. Serpin peptidase inhibitor, clade F, member 1 are able to block apoptosis of immature cerebellar granule cell neurons induced by serum deprivation and K + , and glutamate-mediated degeneration of motor neurons [142, 143]. It was recently found that it is significantly reduced in EVs of MDD patients, which triggers aberrant Wnt signal and synaptic damage, thereby inducing depressive-like behavior [144, 145]. The ratio of brain-derived neurotrophic factor brain derived neurotrophic factor(BDNF) and pre-BDNF can monitor the therapeutic progression of psychiatric disorders. They are difficult to cross the BBB and have a low half-life in serum, but it can be avoided by BDNF and pre-BDNF on EVs [146–149]. Gelle et al. [150], demonstrated an increase in BDNF and a decrease in pre-BDNF in EVs, during antidepressant treatment for MDD, but there was no significant correlation between changes in two of neurotrophic factors, clinical improvement and depression scales in this study. An expanded sample size and clinical parameters may be needed to further investigate the relationship between differential expression of those and depression. Nasca et al. [151], found that high levels of insulin receptor substrate-1 in EVs that rich in L1 cell adhesion molecular, are associated with psychiatric disorders, such as guilt and suicide, and their relationship may provide evidence for a metabolic subtype of depression. The main molecular mechanism may be the accumulation of insulin receptor substrate-1 in EVs leading to reduced insulin receptor binding sensitivity and disruption of insulin signaling [152, 153]. Schizophrenia always has lacked reliable biomarkers to detect an accurate classification. The overabundance of miR-137 and little cytochrome c oxidase subunit VIa polypeptide2 in EVs corresponds to the reduced gamma-band auditory steady-state responses oscillations was found in the early schizophrenia patient model due to mitochondrial dysfunction–parvalbumin interneurons damage [154]. This implies that changes in the concentrations of miR-137 and cytochrome c oxidase subunit VIa polypeptide2 in EVs are associated with disruption of parvalbumin interneurons cortical microcircuits, which could help identify early schizophrenia patient caused by mitochondrial dysfunction.

In addition, microglia-derived EVs containing mir-146a-5p regulate neuronal function in the dentate gyrus and inhibit neurogenesis and spontaneous firing via the mir-146 A-5p/krüppel-like factor 4 pathway [155, 156]. Reducing or blocking the secretion of miR-146a-5p in EVs improves neurogenesis deficits and depression-like behavior in adults. Wei et al. [157], found that EVs extracted from the blood of MDD patients contain miR-139-5P that induces depression-like behavior in normal mice. miR-139-5p, a negative regulator of neural stem cell proliferation and neuronal differentiation, is upregulated in a chronic unpredictable mild stress mouse model and leads to impaired neurogenesis [157]. Interestingly, EVs derived from the blood of patients with depression do not only have negative effects. Another study [158] suggests that in a lipopolysaccharide-induced inflammation model, the expression of Sigma-1 receptors is upregulated in EVs from the blood of patients with depression. This upregulation enhances the production of BDNF, alleviating depression-like behavior and neuroinflammation. In fact, the findings of Wang [158] is not contradictory to Wei et al. [157], but rather indicates that EVs from the blood of patients with depression carry both pathogenic and reparative factors. However, depending on the type of depression, there may be significant differences in the concentrations of functional factors carried by blood-derived EVs, resulting in variations in the functions of EVs from patients with depression, such as miR-139-5p and Sigma-1.

In addition to blood-derived EVs, Other cell-derived EVs also can be used as potential drugs to treat depression. MiR-207 in NK cell-derived EVs interferes the signal conduction of nuclear factor kappa-B in astrocyte by acting on TLR4 and reduce the concentration of pro-inflammatory cytokines containing IL-1β, IL-6 and TNFα [159]. BMSCs-EVs upregulate miR-26 and ameliorate damage of hippocampal neurons in depressed rats [160]. MiR-26a overexpression can increase the level of superoxide dismutase and reduce malondialdehyde, lactate dehydrogenase, TNF-α and IL-1 β levels to promote cells proliferation in hippocampus [160].

## Huntington’s disease

HD is an autosomal dominant disease of the cerebral cortex for mutant huntington (mHTT) accumulation in cells [161, 162]. Lee et al. [163], shared circulation with young (mouse) blood via heterochronic parabiosis reduces mHTT aggregation, improves mitochondrial dysfunction, and restores cognition in a mouse model of HD. This study validates the ability of EVs in young blood to improve HD and also clearly indicates that EVs are the messenger units that deliver positive factors in the blood. Insulin-like growth factor 2 as one of the positive factors was found to increase the secretion of soluble mHTT protein through EVs’ transmission, which was able to reduce the accumulation of abnormal proteins [164]. This result provides indirect evidence for treating HD by genetic modification that engineering EVs with insulin-like growth factor 2. EVs from different sources carry different HD-related positive factors. EVs derived from adipose stem cells provide neuroprotection in HD by activating the p-CREB-PGC1a pathway [165]. EVs carrying miR-124 inhibit HD progression by supporting neurogenesis, upregulating neurotrophic factors, and promoting neuronal differentiation [166–168]. Silencing mHTT by specifically delivering siRNAs so as to prevent the expression of proteins related to the disease phenotype, might be a greater ideal strategy. EVs loaded with hydrophobically modified siRNAs silence Huntington mRNAs, and decrease the accumulation of mHTT in a dose-dependent manner [169]. A recent synthetic biology strategy combines the EV-circulating system with artificial genetic circuits. A cytomegalovirus promoter-directed genetic circuit was constructed to produce RVG tags and mHTT siRNAs upon uptake by the liver [170, 171]. The naked DNA plasmids, in the form of genetic circuits, were intravenously injected into the liver to express transgenes. These transgenes direct the self-assembly and secretion of EVs containing RVG and mHTT–siRNAs. Subsequently, the mHTT siRNAs loaded RVG–EVs are specifically targeted into the cortex and striatum through the EVs-circulating system [170]. The pathological protein levels in the cortex and striatum of the HD significantly reduced, thus buffering the lesion damage in brain and improving behavior score.

## Conclusion

MRI and other large devices are costly, and spinal fluid punctures are invasive, making monitoring the progress and treatment of CNS diseases challenging. EVs, stable carriers of protein, mRNA, miRNA, and lipids in bodily fluids, prove valuable for evaluating such diseases [172]. Brain-derived EVs effortlessly cross the BBB, promoting intercellular communication to regulate CNS homeostasis or activate cytotoxic responses in receptor cells [173]. Therefore, the quantity, properties, and composition of EVs can be utilized for the early diagnosis of CNS diseases. In addition, EVs can deliver bioactive substances through various pathways, safely and efficiently transferring these substances to participate in cellular metabolism. Leveraging the absorption mechanisms of EVs in cells enhances the efficiency of transporting nucleic acids, enzymes, and small-molecule drugs [174]. EVs, ideal for transporting molecular drugs, have found widespread application in studying vascular diseases, malignant tumors, neurodegenerative disorders, psychiatric conditions, and genetic diseases, providing new strategies for treating neurological disorders. The EVs imaging opens up a new frontier for understanding CNS diseases, presenting an opportunity for combining imaging and drug therapy in diagnosis and treatment. However, clinical research on EVs for the diagnosis of CNS diseases is still in the experimental stage. There is a lack of uniform standards for EVs in assessing CNS diseases, and there is insufficient support from large multicenter samples. Many practical challenges need to be overcome for the clinical translation of EVs as effective biomarkers for neurological diseases. Despite these challenges, their potent advantages as diagnostic tools remain significant. As larger scale studies on EVs from clinical samples are conducted, standards based on EVs as biomarkers for CNS diseases are expected to become more detailed and reliable.

Furthermore, the clinical application of EVs still faces numerous limitations, such as low production yield, low drug dosage, and precise targeting. These challenges are the primary obstacles to the clinical use of EVs. Recent studies have shown a significant increase in EVs derived from three-dimensional culture compared to conventional two-dimensional culture [175]. Current research efforts focus on enhancing EVs production, drug loading capacity, and improving targeting. Future studies on EVs are likely to prioritize increasing drug delivery efficiency and EVs yield. The application of EVs in the CNS requires more evidence, including optimal dosage, measurement standards, and the best administration routes for EVs therapy [176]. Dosing strategies and efficacy in small rodent models and large primates cannot be lumped together, posing a long-term challenge to the clinical translation of EVs research. Collaborative efforts from the academic community, clinical medicine, and various institutions are essential for overcoming these challenges [177]. Nonetheless, with the continued development of EVs nanotechnology, The early diagnosis and treatment techniques for CNS diseases are poised to enter a new era.Nerve.

## Data Availability

Data availability is not applicable to this article as no new data were created or analyzed in this study.

## Abbreviations

EVs: Extracellular vesicles
CNS: Central nervous system
BBB: Blood–brain barrier
PD: Parkinson’s disease
HD: Huntington’s disease
AIS: Acute ischemic stroke
α-syn: α-Synuclein
MDD: Major depressive disorder
BDNF: Brain derived neurotrophic factor
SPION: Superparamagnetic iron oxide nanoparticle
MRI: Magnetic resonance imaging
FTH1: Ferritin heavy chain
BMSCs: Bone marrow-derived mesenchymal stem cells
Cur: Curcumin
GNP: Gold nanoparticles
RVG: Rabies virus glycoprotein
c(RGDyK): Cyclo(Arg–Gly–Asp–D-Tyr–Lys) peptide
mAb–GAP43: Monoclonal antibody against GAP43
ROS: Reactive oxygen species
IL-1β: Interleukin-1β
IL-6: Interleukin-6
TNF-α: Tumor necrosis factor-α
mHtt: Mutant huntington
